# Understanding adaptive teamwork in health care: Progress and future directions

**DOI:** 10.1177/1355819620978436

**Published:** 2020-12-16

**Authors:** Janet E Anderson, Mary Lavelle, Gabriel Reedy

**Affiliations:** 1 Professor of the Quality of Care for Older People, School of Health Sciences, City University of London, UK; 2 Senior Research Fellow, School of Health Sciences, City University of London, UK; 3 Reader in Clinical Education, Faculty of Life Sciences and Medicine, King’s College London, UK

**Keywords:** interprofessional teamwork, adaptive teams, research design

## Abstract

Health care teamwork is a vital part of clinical work and patient care but is poorly understood. Despite poor teamwork being cited as a major contributory factor to adverse events, we lack vital knowledge about how teamwork can be improved. Teams in health care are diverse in structure and purpose, and most patient care depends on the ability of different professionals to coordinate their actions. Research in this area has narrowly defined health care teams, focused mainly on a small range of settings and activities and addressed a limited range of research questions. We argue that a new approach to teamwork research is needed and make three recommendations. First, the temporal and dynamic features of teamwork should be studied to understand how teamwork unfolds sequentially. Second, contextual influences should be integrated into study designs, including the organization of work, tasks, patients, organisational structures, and health care system factors. Finally, exploratory, rather than confirmatory, research designs are needed to analyse the complex patterns of social interaction inherent in health care work, to build our theoretical understanding of health care teams and their work, and ultimately to develop effective interventions to support better teamwork for the benefit of patients.

## Introduction

There is growing awareness that health care systems are complex, open, and adaptive,^1^ with emergent effects that are difficult to predict and control. In such a rapidly evolving environment teams must respond flexibly to emerging problems moment-to-moment,^2,3^ but our understanding of how teams coordinate their efforts to respond flexibly is not well developed. Research has identified some of the characteristics of effective teams in fields such as psychology and management, but it is only recently that attention has been paid to investigating the dynamic features of teams, and their adaptative capacity. Even less attention has been paid to adaptive teams in health care settings.

Adaptive capacity is defined as the ability to coordinate activities under routine and novel conditions, which requires the ability to respond to situational requirements.^4^ Recent work in this area has shown that the ability of the team to coordinate an effective response to environmental changes is crucial to performance,^5,6^ but more questions need to be addressed to guide the design and ongoing management of adaptive teams. This gap in knowledge is especially problematic because poor teamwork is one of the most cited contributory causes of adverse events^7^ and should therefore be a priority for improvement efforts. Embracing the importance of adaptive capacity in health care teams means that new theories and methods are required to understand the highly nuanced social interactions that characterize the complexity, variability, and emergent nature of health care teamwork.

In this paper, we argue that there is a need to shift the focus of research efforts and to think more broadly about how best to study dynamic, complex phenomena such as teams of health professionals engaged in patient care. Instead of viewing teamwork as an intervention with a linear and predictable effect on patient outcomes, we need to understand the intricate sequences of adaptive behaviours involved in coordinating a diverse group of health professionals to respond to changing priorities and challenges. This will require a different approach to teamwork research, which we articulate. We recommend four fundamental changes in teamwork research to advance this agenda.

## Health care teamwork

Teamwork can be defined as work that requires the coordination and articulation of tasks and activities between groups of people.^8^ Depending on the health care setting and the tasks involved, clinical work might not occur in formally constituted teams with shared objectives and performance measures, leading some researchers to question whether this can be called teamwork.^9^ Yet such work still requires the articulation and coordination of different activities, even those occurring asynchronously and requiring communication between many people. Therefore, we argue that teamwork should be defined broadly in health care and must include activities which rely on effective coordination between people who may not meet formal definitions of a team, but who are nevertheless required to work effectively with others. In accord with others,^10^ we propose a broad definition based on the different inter-professional activities required for safe and effective care. Such a definition would allow researchers to focus on the full range of coordination activities, consider the full range of team types in health care, and identify how to support coordination activity in all its forms.

It is well recognized that teamwork in health care is difficult. Team members may work in different spaces, work different hours, and come from different professional backgrounds with different training, knowledge, attitudes, and expectations. Turnover of team members is typically high, and when combined with shift work, this means that team members do not always know each other or appreciate the competencies of the people they are working with. Moreover, power hierarchies within and between professions may operate to hinder junior staff, or whole professional groups, from participating as full team members.^11^ Patients and family members are often not recognized as members of the team, despite their crucial roles in the care process. These features of health care teamwork demand more focused research efforts to understand the challenges and devise solutions.

Despite the proliferation of many different teamwork theories and frameworks, few have responded to these unique features or the diversity of teamwork structures encountered in health care, drawing instead on theories and definitions developed in other domains. Frameworks have been transferred from other domains such as aviation and military missions,^12^ which often have well-defined goals and tasks and stable team membership. Consequently, teamwork research in health care has focused on areas with similar characteristics, such as surgery and emergency care,^13^ even though they represent only a small proportion of health care work.

Our recent research on ward teams identified five different types of teams: structural, hybrid, satellite, responsive and co-ordinating. Each team type had a different structure and purpose, and experienced different challenges that required them to adapt in different ways to maintain effective performance.^14^ These findings strongly support the need to conduct research that is sensitive to the team type, structure, and context.

## Limitations of current research

We argue that current research has several limitations that have hindered the accumulation of knowledge that would inform practice. First, there has been an emphasis on the summative assessment and evaluation of teamwork, often using surveys and structured observational tools.^15^ Such cross-sectional methods allow only limited insights into the complex choreography of adaptive teamwork. Teamwork is emergent and dynamic as team members react to each other’s words and behaviour and to the demands of the environment. We need to use tools and methods for understanding this complex choreography as it unfolds, such as *in situ* observations^16^ and detailed analysis of interactions.

Second, outcomes of teamwork interventions are often defined as improvements in patient care,^17^ with relative neglect of a range of other important process outcomes. Evidencing the impact of teamwork interventions on patient care in a complex system where there are multiple proximal and distal factors affecting care quality is difficult, especially if there are small numbers of people involved, which often precludes statistical analysis. We need to include process measures, such as changes in teamwork behaviours, adaptive response behaviours, and evidence of team culture and climate that can enrich our understanding of how teamwork leads to clinical outcomes. Although some studies have used such evaluation measures,^15^ these have often been part of a randomized controlled trial and based on structured surveys and observational tools that do not account for the dynamic and emergent nature of teamwork as it evolves. We argue that, although these approaches give some insight into teamwork, there remains a need to include different study designs and evaluation measures, which focus on the dynamic and emergent aspects of teamwork.

Third, input-process-output (IPO) models, which have been influential in teamwork research, are limited in their ability to uncover the temporal and dynamic features of teamwork, such as adaptive performance.^18^ Research in this tradition has established clear links between teamwork and other variables, including patient outcomes, different approaches to team training, and some measures of team effectiveness. It has also helped to identify the main competencies required for effective teams. While this has informed our understanding of teams, it remains limited for understanding the complex dynamics of adaptive teams. Effective health care teams must flexibly respond to the changing demands of health care environments, resolve competing goals, prioritize their actions, and co-ordinate their efforts.^19^ Complex multi-layered interactions and feedback loops characterize adaptive teamwork, and linear IPO models do not adequately reflect this.^20,21^

Fourth, teams are often treated as closed systems with their performance being determined by team factors whereas contextual factors are not given prominence. Although IPO models acknowledge the importance of some factors, such as team member knowledge, it is arguably just as important to understand the wider context, including the influence of work organization, task characteristics, culture, and health care system factors. These factors are likely to be important in shaping teams and must be understood if the goal is to design and maintain effective teams.

Finally, positivist research designed to identify objective, evidence-based, replicable interventions may limit our ability to understand the complexity of interactions between and within teams and their environments. Study designs that are relevant for evaluating the effectiveness of medical treatments, such as randomized controlled trials, are not appropriate for the study of complex social interactions, or for understanding complex behaviour, such as how teams adapt flexibly to situational demands. To advance our knowledge and understanding, we need to use the best research approaches for answering these questions. Several recent reviews support these limitations, having identified the need to integrate knowledge from different disciplines; the value of using nonlinear, non-positivist research designs to understand dynamic and emergent phenomena over time; and the need to contextualize teamwork by understanding the tasks, pressures, organizations, and multiple team structures within which teams operate.^18,20,21^

## Reframing health care teamwork research

The conceptual and philosophical underpinnings of research are frequently not explicated, but in arguing for a new approach to the research and scholarship of teamwork in health care, we believe it is important to articulate exactly how a new approach requires a different philosophical framework. Epistemological assumptions are related to ontological beliefs about the nature of the phenomena being studied, and together they are linked to specific methodological approaches and methods: thus, articulated or not, they shape every detail of study design.^22^ To develop new theories and methods, we therefore need to articulate and disambiguate these assumptions and beliefs.

By analysing existing teamwork research according to its design, one could argue that the unarticulated epistemological assumptions of much teamwork research are that health care work proceeds in a linear fashion that can be specified in policies and procedures, and that teamwork can be measured objectively and can be ‘treated’ to correct its deficiencies. Moreover, simplified concepts of work imply that teamworking interventions can be standardized and introduced without difficulty, and that their effects on patient care can be traced and quantified. Such linear thinking is not helpful for understanding and influencing complex social structures, not least because it assumes that we can specify in advance exactly how people should and will respond in any given situation.

Such assumptions also do not accord with recent new understanding of health care systems that emphasize complexity, emergence, and adaptation as core features of the work.^23^ Just as these insights require new approaches to quality improvement,^3^ they also require new approaches to teamwork research. Table 1 summarises different philosophical assumptions underpinning teamwork research, contrasting positivist and constructivist paradigms, and describes the ontology, epistemology, methodology, methods, and research questions associated with each.

**Table 1.** Contrasting philosophical assumptions underpinning teamwork research.

Paradigm	Ontology: what is the nature of teamwork?	Epistemology: how can knowledge about teamwork be produced?	Example research questions	Methodology	Methods
Positivist	• Deterministic: optimal behaviour can be specified, causal relationships can be established• Teamwork is stable over time and place • Teamwork can be objectively defined and measured	• General laws about team behaviour can be objectively established• Interventions can be standardized • Interventions have a linear effect on outcomes	• How effective is the teamwork? • What interventions improve teamwork? • What team interventions improve patient care? • What is the most effective way to train teams to work together?	• Confirmatory: hypothesis testing, summative assessment	• Measurement using rating scales and validated tools• Evaluation of outcomes • Randomized controlled trials• Quasi-experimental designs
Constructivist	• Emergent: teamwork is highly contingent • Teamwork cannot be understood in isolation from task pressures and challenges • Teams must adapt to changing situations and reprioritize goals• Teams are social entities	• Knowledge involves subjectivity• Contextual, environmental, team and organisational factors are important so teams should be studied in situ• Teams evolve and should be studied over time	• How do task, contextual and organisational factors combine to affect teamwork? • What team skills are needed to adapt to pressures and problems? • How do teams orchestrate and coordinate their actions to achieve outcomes? • What aspects of the work system challenge teams’ ability to co-ordinate their actions? • How does teamwork change over time?	• Exploratory: analysis of patterns, video and audio data, analysis of context	• Interaction analysis• Behavioural analysis• Qualitative methods • *In situ* observation

Note: The ontology-epistemology-methodology-method framework used in the table has been referenced by many qualitative researchers.^22,24^ The teamwork content and proposed questions were developed by the authors.

In practice, these two paradigms and the mapping of methods to research questions may be more nuanced than presented here, and there is likely to be some overlap. However, our argument is that there has been relative neglect of the research questions associated with the constructivist paradigm, and that this has limited our collective understanding of health care teams. It is therefore helpful to disambiguate the two positions. Articulating the underlying assumptions can raise awareness of different approaches, broaden the research questions investigated, and inform researchers who may be unfamiliar with different research traditions.

## Future directions for research

As shown in Table 1, we argue that a different ontological-epistemological perspective is required to advance health care team research, based on the constructivist paradigm. This has four related implications for teamwork research.

First, adaptive teamwork should be viewed as an emergent, dynamic social interaction in a complex environment, and the focus should be on understanding how teamwork emerges, is sustained, and changes over time and how it can be supported. The focus on competency-driven education in health care has resulted in the use of cross-sectional study designs to evaluate teamwork, which are limited in their ability to understand dynamic adaptive behaviours. We need to focus on the dynamic and temporal features of team interaction, and to use process measures in addition to outcome measures. It is only by understanding this complex choreography as it unfolds temporally that we can produce the knowledge to inform teamwork training, team design, and organisational support for teamwork.

Research in disciplines such as human factors,^25^ organisational psychology^18^ and management science^26^ has provided important insights, frameworks and techniques that acknowledge the complexity and dynamism of teamwork. A new approach in health care could draw on this work and develop it for the benefit of health care team research.

Second, contextual factors should be integrated into teamwork models and theories. Contextual factors, as they become a focus of exploration in a new approach to teamwork research, include such things as different team structures, clinical demands, tasks, and organisational structures and processes. There is currently little understanding of how contextual factors affect team adaptation. For example, it is likely that different types of pressures, such as resource limitations, information uncertainty, provision of new information, changes in organisational performance goals, and patient needs and preferences^5,19^ require different adaptive responses, but this has received relatively little attention.

Third, to address the above points, we require exploratory rather than confirmatory research designs.^27^ Such study designs are invaluable when effectiveness studies are either not feasible or appropriate, and when research questions are more nuanced. The goal would be to explore and identify the complex sequences of interaction that unfold over time when workers coordinate their actions to achieve a goal, rather than only testing hypotheses using experimental designs. Although each team interaction is in some senses unique, the research focus should be on identifying overarching patterns of interaction that can be understood in theoretical terms. This requires theoretically informed exploration to identify patterns of behaviour and their functions, grounded in a deep understanding of the context.

Exploratory research designs should not be judged by applying indicators of rigour used in confirmatory research designs, such as large sample sizes, external validity, and effectiveness outcome measures. Sequential data such as audio or visual recordings of team interactions are usually composed of episodes of interaction which are analysed in detail and then aggregated. The resources and effort required to analyse episodes of interaction are significant, and, depending on the grain of analysis, can result in hundreds of behaviours identified in just a few minutes of interaction. Since pattern exploration is the goal, it is often more important to ensure there is a large enough sample of behaviours than to maximize the sample of episodes. External validity may have less importance if the goal is to integrate context into the analysis. Detailed description of work systems and scenarios can also provide a basis for generalisability claims. This is especially true if the research is problem-driven, as suggested here, rather than searching for definitive and universal aspects of human behaviour.^28^ Effectiveness outcome measures, such as quality of care, are often precluded in exploratory research because the depth of analysis and small numbers of team members prevent inferential statistical analysis. Moreover, team outcomes are important process measures that should not be neglected.

We are heartened that many promising studies are now beginning to emerge that respond to the need for which we have argued here: namely, to study team interaction and context in depth.^16,25^ By highlighting the limitations of the field in general, we hope to encourage more research that adopts the approach we advocate.

## Conclusions

Health care teamwork research has narrowly defined the nature of health care teams, focused mainly on a small range of settings and activities and addressed a limited range of research questions. We propose that teamwork should be broadly defined and argue that the underlying assumptions about the nature of teamwork and how it should be studied have shaped, and limited, research. We here propose three elements of a new approach based on a constructivist paradigm that is needed to progress the field. First, the dynamic and emergent aspects of adaptive teams should be studied to understand how teamwork unfolds sequentially in response to the environment. Second, contextual influences should be integrated into study designs, including the organization of work, tasks, patients, organisational and health care system factors. Finally, exploratory, rather than confirmatory, research designs are needed to analyse the complex patterns of social interaction inherent in health care work, build our theoretical understanding of health care teams and their work and ultimately lead to effective interventions to support better teamwork for the benefit of patients.
